# How Do Bacteria Know They Are on a Surface and Regulate Their Response to an Adhering State?

**DOI:** 10.1371/journal.ppat.1002440

**Published:** 2012-01-26

**Authors:** Henk J. Busscher, Henny C. van der Mei

**Affiliations:** Department of Biomedical Engineering, W.J. Kolff Institute, University Medical Center and University of Groningen, Groningen, The Netherlands; University of North Carolina, United States of America

## Why Do Bacteria Adhere to Surfaces and Why Is Adhesion Considered a Virulence Factor?

Bacteria adhere to virtually all natural and synthetic surfaces [1], [2]. Although there are a number of different reasons as to why bacteria adhere to a surface, the summarizing answer is brief: “Adhesion to a surface is a survival mechanism for bacteria”. Nutrients in aqueous environments have the tendency to accumulate at surfaces [1], [3], giving adhering bacteria a benefit over free floating, so-called planktonic ones. This is why mountain creeks may contain crystal clear, drinkable water, while stepping stones underneath the water may be covered with a slippery film of adhering microbes. In the oral cavity, adhesion to dental hard and soft tissues is life-saving to the organisms, because microbes that do not manage to adhere and remain planktonic in saliva are swallowed with an almost certain death in the gastrointestinal tract.

Bacterial adhesion is generally recognized as the first step in biofilm formation, and for the human host, the ability of a bacterium to adhere is a definite virulence factor, especially in immunocompromised patients and in the growing number of elderly patients relying on biomaterials implants and devices for the restoration of function after (oncological) intervention surgery, trauma, or wear [4]. Well-known examples of biomaterials implants are dental implants, vascular grafts, and prosthetic hips and knee joints. Bacterial adhesion is a virulence factor, because it stimulates the organism to produce extracellular polymeric substances (EPSs), such as polysaccharides, proteins, nucleic acids, and lipids [5], through which they embed themselves in a protective matrix. This protective matrix provides mechanical stability to a biofilm and constitutes the main difference between planktonic bacteria and bacteria adhering to a surface in a so-called biofilm mode of growth. Bacteria organized in biofilms are at least ten to 1,000 times more resistant to antibiotics [6] than bacteria in a planktonic state and can cope much better with unfavorable external conditions in the host immune system than their planktonic counterparts. Not surprisingly, the fate of an infection associated with a biomaterials implant is mostly removal and replacement of the implant [7], at high costs to the health care system and great inconvenience to the patient.

## What Are the Mechanisms Controlling Bacterial Adhesion to Surfaces?

Over the past decades, two mechanisms have been described to control microbial adhesion to surfaces. From a biochemical perspective, bacterial adhesion has been described in terms of specific interactions between localized, specific molecular groups. For example, *Escherichia coli* expresses type 1 fimbriae possessing tip-positioned adhesive protein FimH that bind specifically to mannose [8]. Sometimes, even specific forces have been categorized as a separate class of fundamental interaction forces, although such forces do not exist from a physico-chemical perspective. Adhesion, whether arising from specific, molecular, or non-specific interactions, is supposed to originate from an interplay between ever present attractive Lifshitz-Van der Waals forces, attractive or repulsive electrostatic, hydrogen bonding, and Brownian motion forces [9]. Reconciling the biochemical and physico-chemical perspective [9], [10], specific, molecular interactions are now recognized as highly directional, spatially confined interactions, operative over small distances, arising from highly specific, small stereo-chemical domains on the interacting surfaces, but arising from the above mentioned fundamental physico-chemical forces [10], [11].

## Can We Measure the Forces with which a Bacterium Adheres to a Surface?

Since the introduction of atomic force microscopy [12], it has become possible to directly measure the adhesion forces between bacteria and substratum surfaces [13]. In these measurements, bacteria are attached to a cantilever. Subsequently, the bacterium-coated cantilever is manoeuvred toward a substratum surface, and the force upon approach and retraction of the bacterial probe is recorded from the cantilever deflection. Upon approach, an increasing repulsive force is measured until physical contact, while upon subsequent retraction, an attractive adhesion force is recorded until failure, which is the force value generally reported in the literature as “the adhesion force” [13].

## How Does a Bacterium Know It Is on a Surface?

In the absence of visual, auditory, and olfactory perception, adhering bacteria react to membrane stresses arising from minor deformations due to the adhesion forces felt to make them aware of their adhering state on a surface and change from a planktonic to a biofilm phenotype. *E. coli*, for instance, are known to have mechano-sensitive channels [14].

## How Do Bacteria Respond to Different Adhesion Forces?

**Figure 1:**
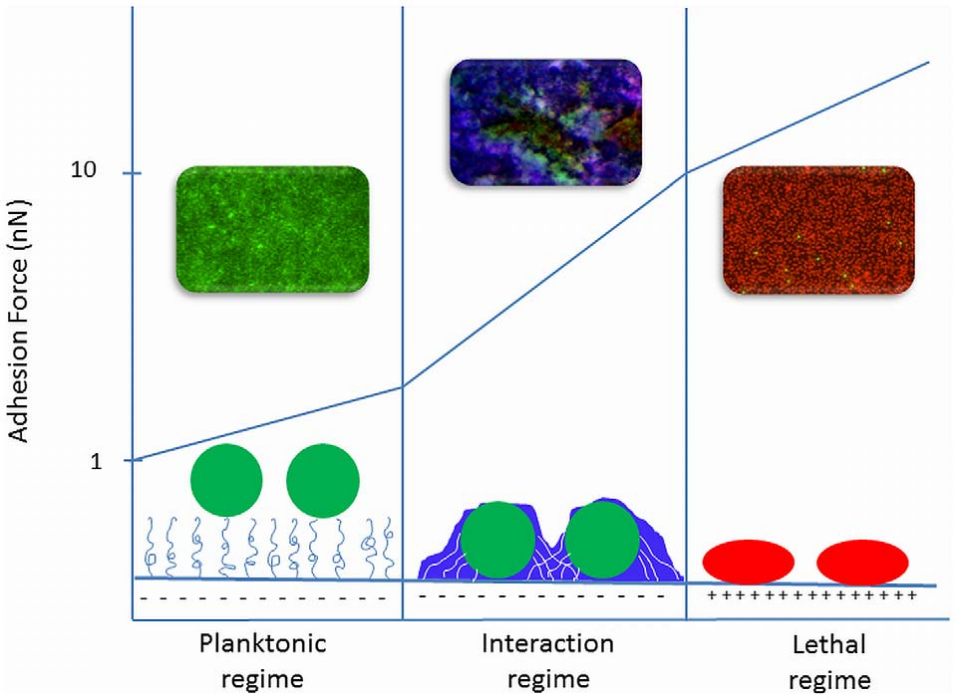
Three regimes of bacterial adhesion to substratum surfaces that dictate the bacterial response to a surface. 1) In the planktonic regime, adhesion forces are extremely weak as on polymer-brush coatings, and bacteria do not realize they are on a surface. Weakly adhering bacteria are mainly live (green fluorescence). This regime is called “planktonic”, because bacteria do not adapt their planktonic phenotype despite their adhering state. 2) In the “interaction” regime, bacterial responses to their adhering state increase with increasing adhesion forces, for instance by the production of EPS (blue fluorescence), encasing themselves in a protective biofilm. 3) In the “lethal regime”, strong adhesion forces, as occurring on positively charged surfaces, cause membrane deformation that causes stress de-activation of the adhering bacteria, leading to reduced growth and cell death (red fluorescence). The confocal laser scanning micrographs represent biofilms in all three regimes of adhesion forces in which bacteria were stained with *Bac*light LIVE/DEAD stain, rendering live bacteria green and membrane damaged or dead bacteria red. EPS was stained with calcofluor white, rendering blue fluorescence.

Based on available literature, we propose three adhesion force regimes dictating the bacterial response to a substratum surface, as schematically summarized in Figure 1. Recently, a link has been described between strong adhesion forces between bacteria and substratum surfaces yielding membrane stresses and the percentage of dead cells on a surface for which the term “stress de-activation” was coined [15]. Stress de-activation may set in gradually with increasing adhesion forces, and in a first instance it has been demonstrated that bacterial generation times on a surface increase with decreasing desorption rates, i.e., increasing adhesion forces [16]. The existence of stress de-activation was further supported by the observation that an external mechanical stress on adhering bacteria enhances the antimicrobial efficacy of quaternary ammonium compounds in solution [17]. Since the great majority of bacterial strains and species possess a negative surface charge [18], strong adhesion forces can be found on positively charged surfaces, such as quaternary ammonium-coated surfaces that are known to kill bacteria upon contact [19] in this “lethal” regime of strong adhesion forces (see Figure 1). It has been suggested that such lethal effects upon adhesion require a minimum positive charge density of the substratum surface [20], [21]; for example, a positive charge density of 8.10^15^ per cm^2^ is required to kill around 10^8^ *E. coli* adhering per cm^2^, equalling a monolayer of bacteria [21]. The positive charge density necessary for lethal effects depends on the bacterial species and is, for instance, ten times higher for *Staphylococcus epidermidis* than for *E. coli* [20].

On the lower end of the adhesion force scale are polymer brush–coated surfaces and hydrogels that exert very weak adhesion forces on adhering bacteria [18] to the extent that adhering bacteria hardly realize they are on a surface and do not change to the protected phenotype enabling them to form a biofilm with EPS encasing [22]. We propose calling this the “planktonic” regime (see Figure 1) of adhesion forces.

In between these two ends of the adhesion force scale is an intermediate or “interaction” regime of bacterial adhesion forces, as encountered on “” materials, such as polymers, metals, and ceramics commonly used for biomaterials implants and devices. In the interaction regime, phenotypic changes occur progressively with increasing adhesion forces. On polyethylene, for instance, with bacterial adhesion forces in the interaction regime, EPS production by adhering staphylococci was much more pronounced than on polymethylmethacrylate or stainless steel [23].

Adhesion forces at the proposed transitions between the different regimes are all approximate because adhesion forces tend to strengthen considerably during the first minutes after contact, yielding a switch from reversible to irreversible adhesion. Microbiologically, this switch has been associated with the production of EPS in response to a surface [2], but EPS production in response to adhesion likely occurs much later on during growth, as completely inert polystyrene particles also demonstrate this initial bond strengthening [24]. Upon first approach of a bacterium to a surface, it becomes attached to a layer of highly viscous water adjacent to the surface that is subsequently slowly penetrated to allow stronger contact with the surface, after which protein structures on the cell surface re-orient themselves to allow optimal binding. Since it is unlikely that metabolic processes and phenotypic changes occur within minutes, we envisage that adhesion forces after physico-chemical strengthening represent the transition forces between the three adhesion force regimes depicted in Figure 1.

The proposal of three adhesion force regimes not only sheds light on how bacteria may sense a surface and what directs their response to a surface, but the implications of these different regimes extend also to interactions between bacteria. Communication between bacteria in a biofilm is often described as being due to quorum-sensing (QS) molecules [2], but it may not be ruled out that the production of QS molecules is also dictated in a first instance by membrane stresses developing as a result of adhesion forces between adhering bacteria in a biofilm in the interaction regime. This suggests two means of bacterial communication in a biofilm: (i) initial signalling through direct physical contact during adhesion to the substratum surface over short distances according to the three regimes of adhesion forces (see Figure 1), and (ii) through QS molecules that diffuse through a biofilm and allow communication over longer distances than possible through adhesion forces, which are limited to several hundreds of nanometers.
